# A second crystalline modification of 2-{3-methyl-2-[(2*Z*)-pent-2-en-1-yl]cyclo­pent-2-en-1-yl­idene}hydrazinecarbo­thio­amide

**DOI:** 10.1107/S2414314623010180

**Published:** 2023-11-30

**Authors:** Adriano Bof de Oliveira, Leandro Bresolin, Vanessa Carratu Gervini, Johannes Beck, Jörg Daniels

**Affiliations:** a Departamento de Química, Universidade Federal de Sergipe, Av. Marcelo Deda Chagas s/n, Campus Universitário, 49107-230 São Cristóvão-SE, Brazil; b Escola de Química e Alimentos, Universidade Federal do Rio Grande, Av. Itália km 08, Campus Carreiros, 96203-900 Rio Grande-RS, Brazil; c Institut für Anorganische Chemie, Rheinische Friedrich-Wilhelms-Universität Bonn, Gerhard-Domagk-Strasse 1, D-53121 Bonn, Germany; Goethe-Universität Frankfurt, Germany

**Keywords:** jasmone thio­semicarbazone, thio­semicarbazone, *cis*-jasmone derivative, crystalline modification, crystal structure, Hirshfeld analysis

## Abstract

A second crystalline modification of the title compound, C_12_H_19_N_3_S [common name: *cis*-jasmone thio­semicarbazone] was crystallized from tetra­hydro­furane at room temperature. There is one crystallographic independent mol­ecule in the asymmetric unit, showing disorder in the *cis*-jasmone chain [site-occupancy ratio = 0.590 (14):0.410 (14)]. The thio­semicarbazone entity is approximately planar, with the maximum deviation from the mean plane through the N/N/C/S/N atoms being 0.0463 (14) Å [r.m.s.d. = 0.0324 Å], while for the five-membered ring of the jasmone fragment, the maximum deviation from the mean plane through the carbon atoms amounts to 0.0465 (15) Å [r.m.s.d. = 0.0338 Å]. The mol­ecule is not planar due to the dihedral angle between these two fragments, which is 8.93 (1)°, and due to the *sp* ^3^-hybridized carbon atoms in the jasmone fragment chain. In the crystal, the mol­ecules are connected by N—H⋯S and C—H⋯S inter­actions, with graph-set motifs *R*_2_^2^(8) and *R*_2_^1^(7), building mono-periodic hydrogen-bonded ribbons along [010]. A Hirshfeld surface analysis indicates that the major contributions for the crystal cohesion are H⋯H (67.8%), H⋯S/S⋯H (15.0%), H⋯C/C⋯H (8.5%) and H⋯N/N⋯H (5.6%) [only non-disordered atoms and those with the highest s.o.f. were considered]. This work reports the second crystalline modification of the *cis*-jasmone thio­semicarbazone structure, the first one being published recently [Orsoni *et al.* (2020 ▸). *Int. J. Mol. Sci.* **21**, 8681–8697] with the crystals obtained in ethanol at 273 K.

## Abstract 2

The crystal structure and Hirshfeld analysis of a second crystalline modification of the *cis*-jasmone thio­semicarbazone is reported. The mol­ecular structure matches the asymmetric unit and the mol­ecules are linked into mono-periodic hydrogen-bonded ribbons along [010].

## Graphical abstract

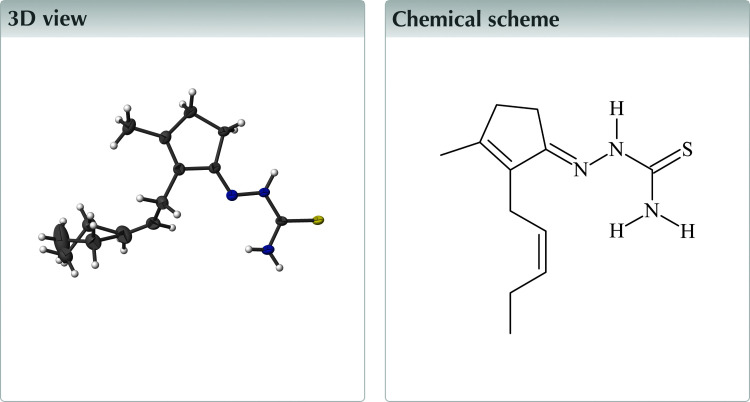

## Structure description

The first references to the synthesis of thio­semicarbazone derivatives [*R*_1_ *R*_2_N—N(H)—C(=S)—N*R*_3_ *R*_4_] can be traced back to the beginning of the 1900s (Freund & Schander, 1902 ▸) and since the report of Domagk *et al.* (1946 ▸) on the tuberculostatic effect of some compounds with this functional group, the biological activity of these mol­ecules has been intensively studied, being one of the major approaches for this chemistry (for some examples, see: Acharya *et al.*, 2021 ▸; Bajaj *et al.*, 2021 ▸; Kanso *et al.*, 2021 ▸; Siqueira *et al.*, 2019 ▸). Concerning the *cis*-jasmone thio­semicarbazone, it has been pointed out that this compound has anti­fungal activity (Orsoni *et al.*, 2020 ▸; Jamiołkowska *et al.*, 2022 ▸). As part of our studies on the thio­semicarbazone derivatives of natural products, the crystal structure and the Hirshfeld analysis of a new crystalline modification of the *cis*-jasmone thio­semicarbazone is reported herein.

The first crystalline modification of *cis*-jasmone thio­semicarbazone (Orsoni *et al.*, 2020 ▸) [triclinic, *P* 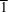 , *a* = 8.164 (5), *b* = 15.645 (9), *c* = 16.434 (9) Å, α = 84.723 (1), β = 82.036 (1), γ = 84.632 (1)°] will be designated from now on as the α-modification and α-JATSC. α-JATSC(A), α-JATSC(B) and α-JATSC(C) abbreviations will be used for the three crystallographically independent mol­ecules in the asymmetric unit of the structure. The present work reports the second crystalline modification of the mol­ecule, which will be designated from now on as the β-modification, or β-JATSC.

**Figure 1:**
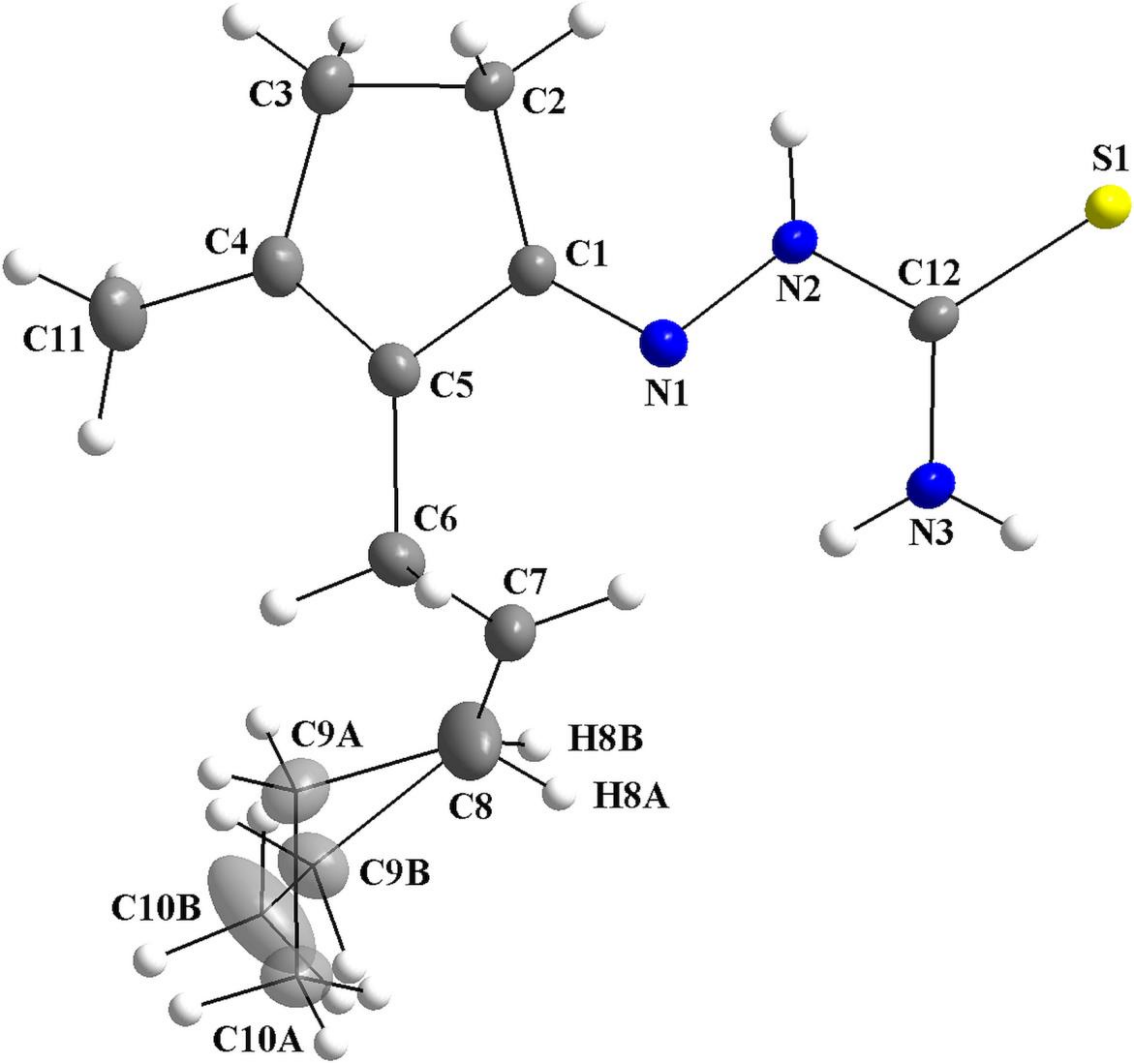
The mol­ecular structure of the title compound, showing the atom labelling and displacement ellipsoids drawn at the 40% probability level. Disordered atoms are drawn with 30% transparency and labelled H8*A*, C9*A* and C10*A* [s.o.f. = 0.590 (14)] and H8*B*, C9*B* and C10*B* [s.o.f. = 0.410 (14)]. All H atoms are drawn in ball and stick mode.

For the title compound, the β-crystalline modification of the *cis*-jasmone thio­semicarbazone, there is one mol­ecule with all atoms in general positions in the asymmetric unit, which shows disorder in the *cis*-jasmone chain [s.o.f. = 0.590 (14):0.410 (14)]. The atoms with the higher s.o.f. are *A*-labelled and those with the lower, *B*-labelled (Fig. 1 ▸). The thio­semicarbazone (**TSC**) entity is approximately planar, with the maximum deviation from the mean plane through the N1/N2/C12/S1/N3 atoms being 0.0463 (14) Å for N2 (r.m.s.d. = 0.0324 Å). The **TSC** entity is attached to the C1–C5 five-membered ring of the jasmone fragment, which is also almost planar, with the maximum deviation from the mean plane through the C atoms being 0.0465 (15) Å for C2 (r.m.sd. = 0.0338 Å). The mol­ecule is not planar due the dihedral angle between these two entities, 8.93 (1)°, and due to the *sp* ^3^-hybridized carbon atoms in the jasmone fragment. In addition, the torsion angles for the N1/N2/C12/S1 and N1/N2/C12/N3 chains are 174.04 (15) and −4.8 (3)°, respectively.

**Figure 2:**
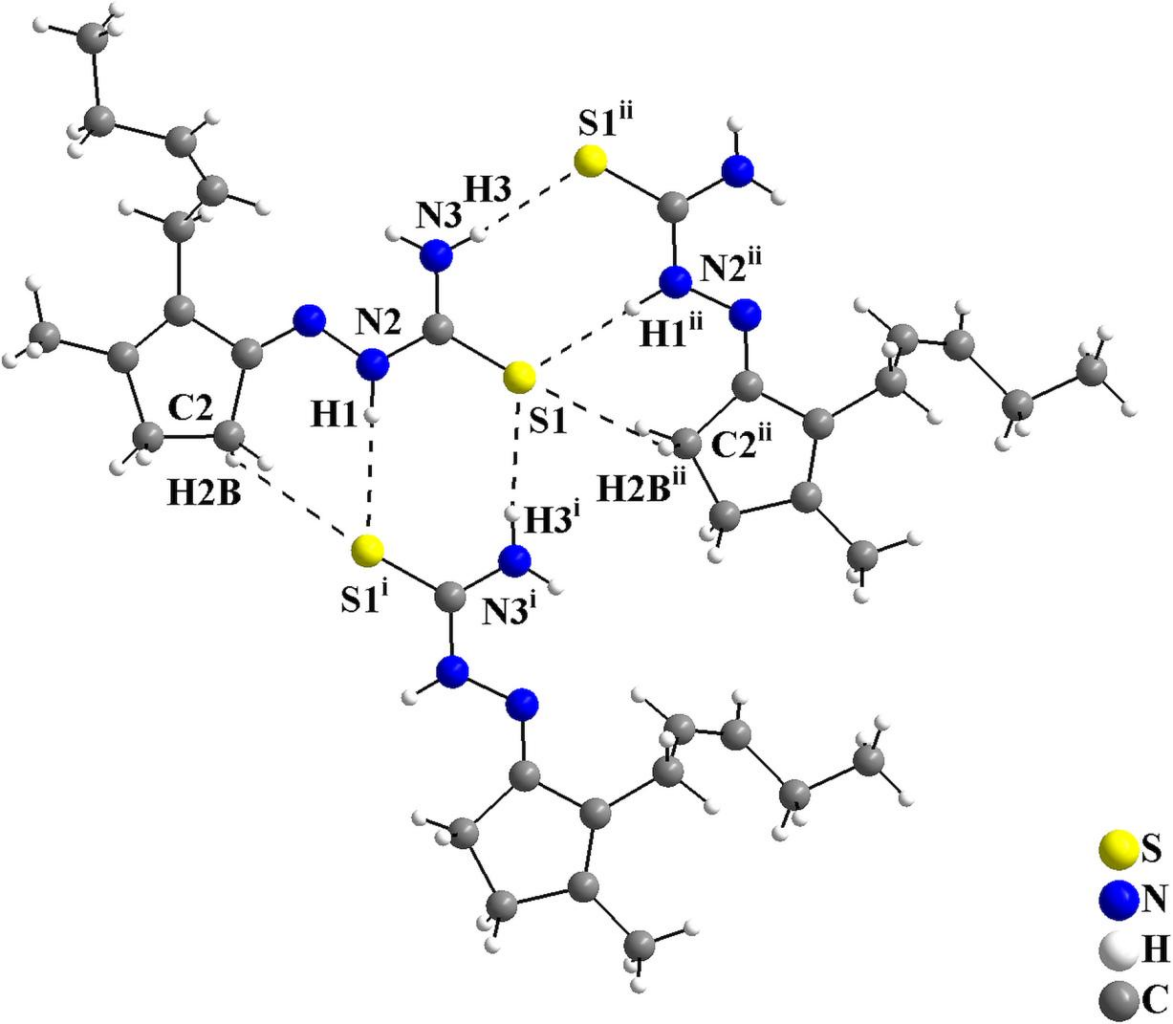
The mol­ecular structure of the β-crystalline modification of the *cis*-jasmone thio­semicarbazone showing the inter­molecular hydrogen-bonding inter­actions as dashed lines. The mol­ecules are linked *via* pairs of N—H⋯S and C—H⋯S inter­actions, forming graph-set motifs of 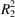 (8) and 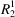 (7). Disorder is not shown for clarity. [Symmetry codes: (i) −*x* + 1, *y* − 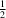 , −*z* + 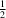 ; (ii) −*x* + 1, *y* + 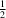 , −*z* + 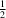 .]

**Figure 3:**
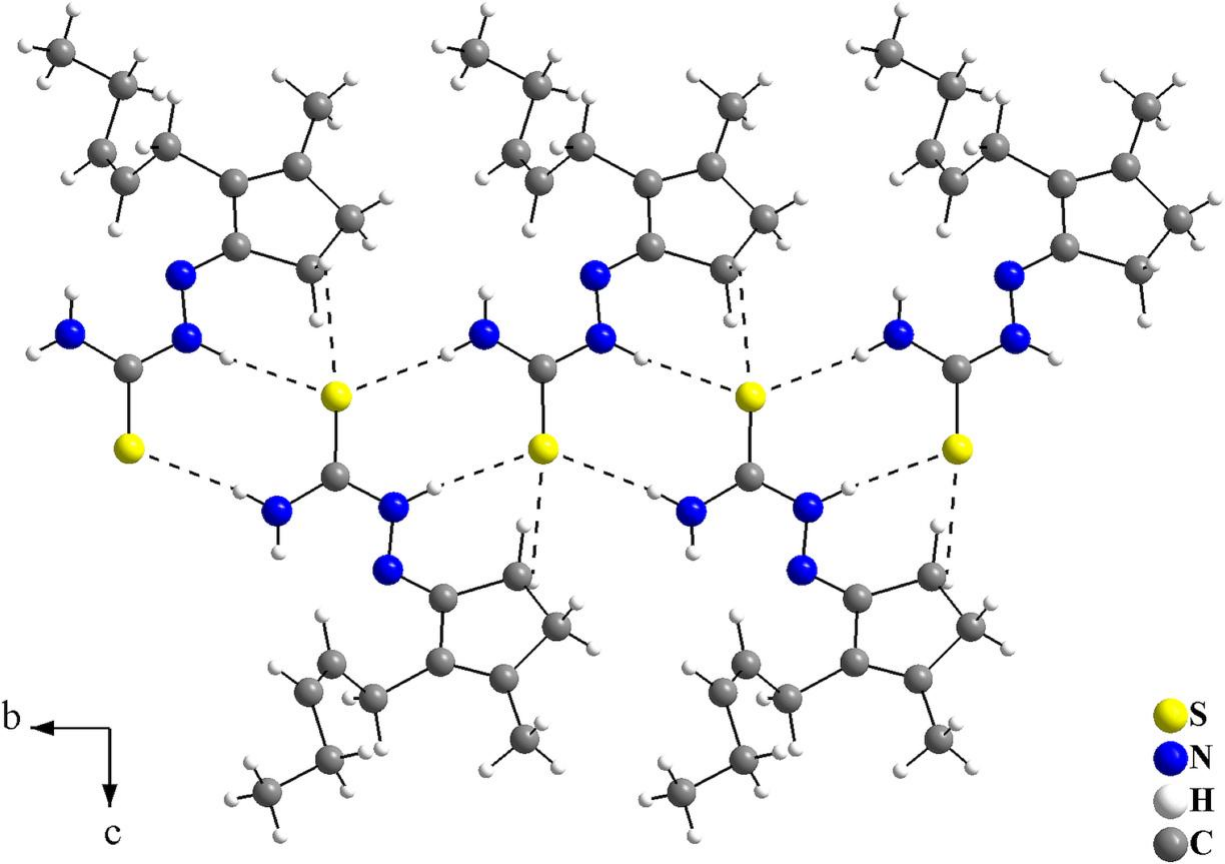
Graphical representation of the N—H⋯S and C—H⋯S inter­molecular inter­actions in the title compound viewed along [100]. The inter­actions are drawn as dashed lines and connect the mol­ecules along [010] with graph-set motifs of 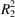 (8) and 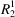 (7), forming a mono-periodic hydrogen-bonded ribbon. Disorder is not shown for clarity.

In the crystal, the mol­ecules are connected by pairs of N—H⋯S inter­actions, forming rings with 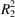 (8) graph-set motif, and by pairs of N—H⋯S/C—H⋯S inter­actions, where rings of graph-set motif 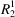 (7) are observed (Fig. 2 ▸, Table 1 ▸). The N1, N3 and C2 atoms act as hydrogen-bond donors and the S1 atoms act as hydrogen-bond acceptors, connecting the mol­ecules into mono-periodic hydrogen-bonded ribbons along [010] (Fig. 3 ▸). No other strong inter­molecular inter­actions are observed for the title compound, possibly due to the non-polar organic periphery of the *cis*-jasmone fragment, and only weak inter­actions, *i.e.*, London dispersion forces can be suggested.

**Figure 4:**
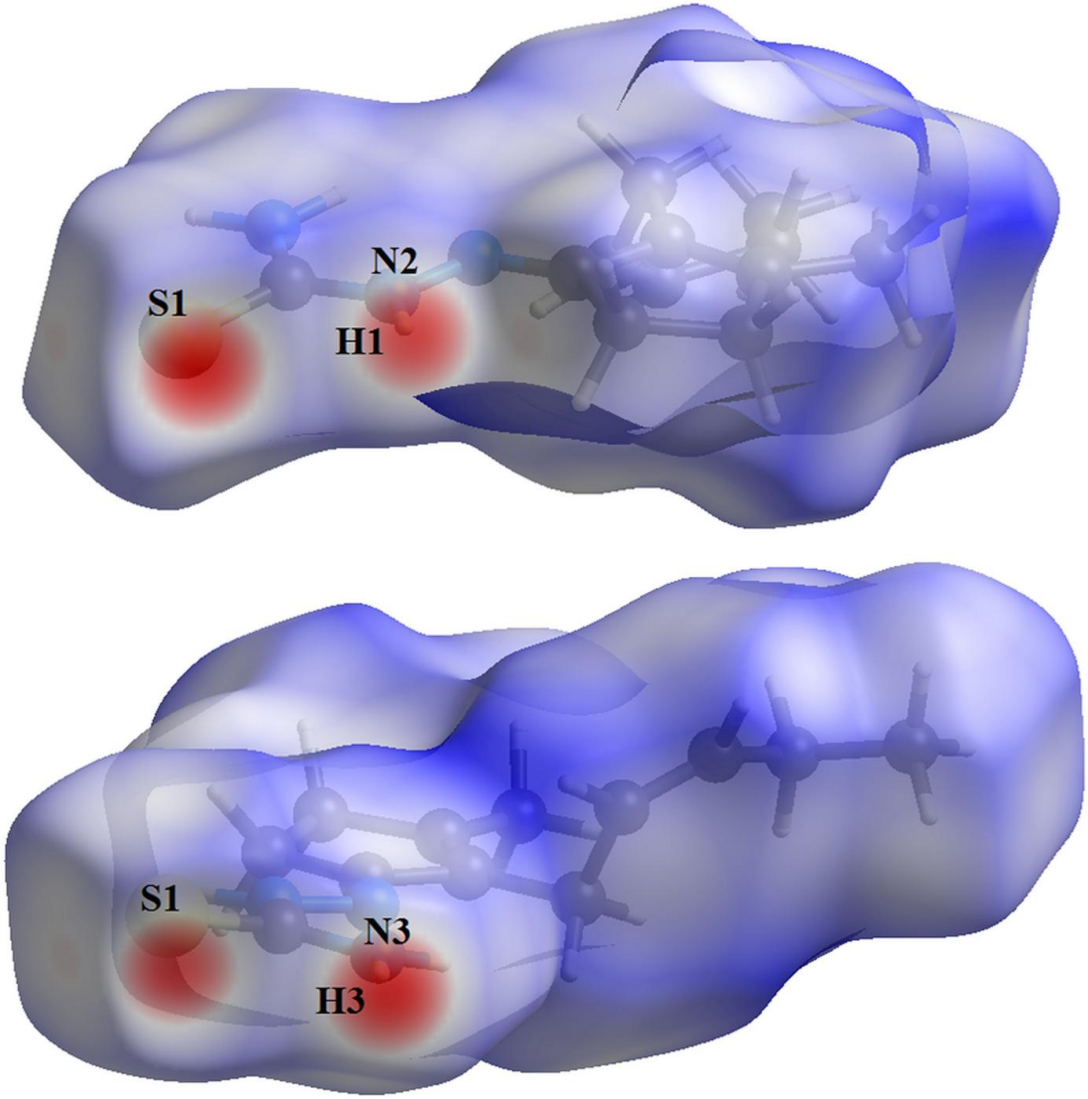
Two opposite side-views in separate figures of the Hirshfeld surface graphical representation (*d*_norm_) for the title compound. The surface is drawn with transparency, the mol­ecule is drawn in ball and stick mode and the disorder is not shown for clarity. The regions with strongest inter­molecular inter­actions are shown in red. (*d*_norm_ range: −0.404 to 1.518.)

**Figure 5:**
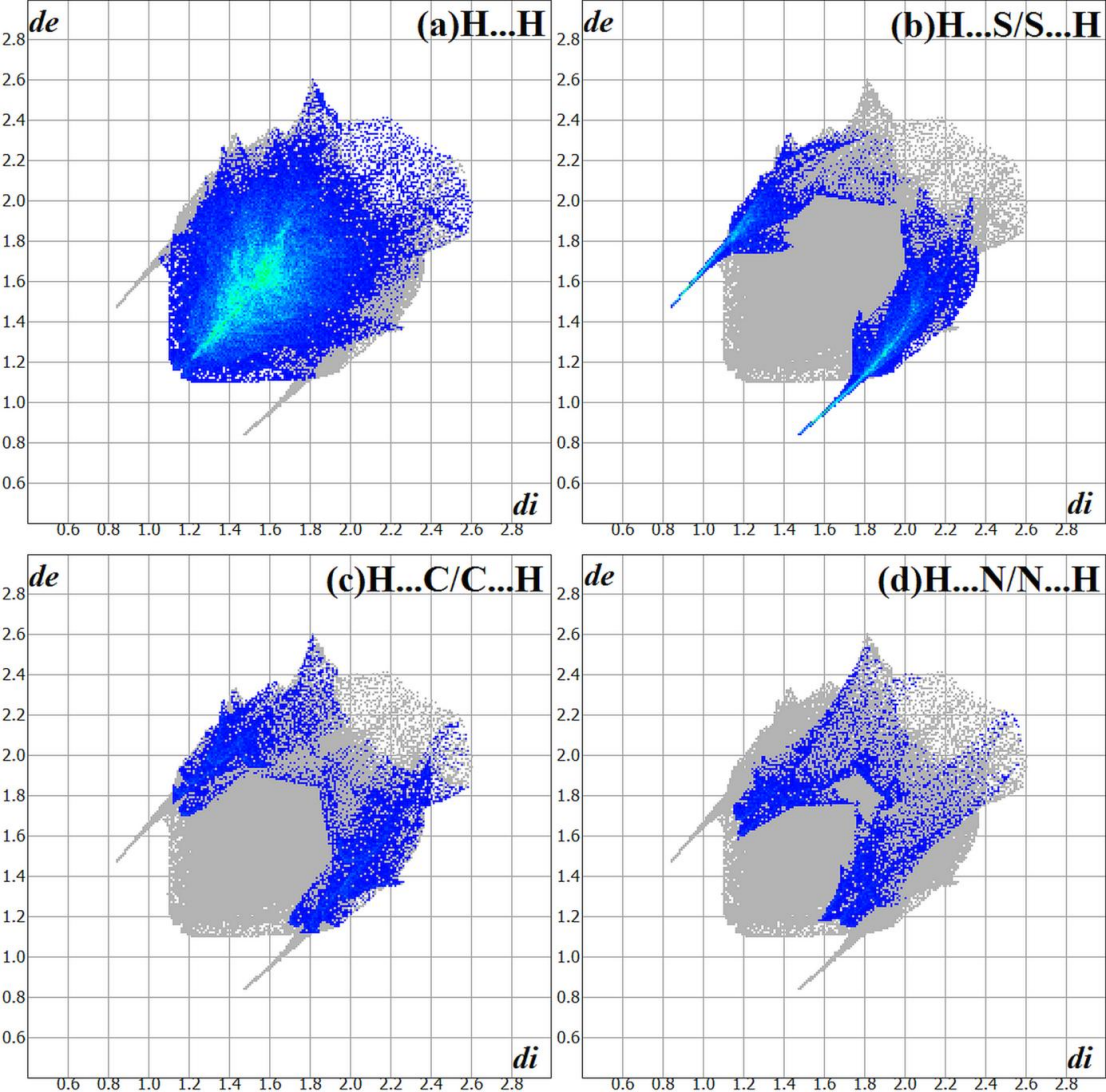
The Hirshfeld surface two-dimensional fingerprint plots for the title compound, showing the contacts in detail (cyan dots). The major contributions of the inter­actions to the crystal cohesion amount to (*a*) H⋯H = 67.8%, (*b*) H⋯S/S⋯H = 15.0%, (*c*) H⋯C/C⋯H= 8.5% and (*d*) H⋯N/N⋯H = 5.6%. The *d*_i_ (*x-*axis) and the *d*_e_ (*y-*axis) values are the closest inter­nal and external distances from given points on the Hirshfeld surface (in Å). Regarding the disorder, only the atoms with the highest s.o.f. were considered.

In the Hirshfeld surface analysis (Hirshfeld, 1977 ▸), the graphical representations and the two-dimensional Hirshfeld surface fingerprint (HSFP) were evaluated with *Crystal Explorer* (Wolff *et al.*, 2012 ▸). The Hirshfeld surface analysis of the title compound considering the *A*-labelled atoms [s.o.f. = 0.590 (14)] indicates that the most relevant inter­molecular inter­actions for crystal cohesion are the following: H⋯H = 67.8%, (*b*) H⋯S/S⋯H = 15.0%, (*c*) H⋯C/C⋯H = 8.5% and (*d*) H ⋯N/N⋯H = 5.6%. For comparison, the contributions for the structure with the *B*-labelled atoms [s.o.f. = 0.410 (14)] amount to (*a*) H⋯H = 68.3%, (*b*) H⋯S/S⋯H = 15.0%, (*c*) H⋯C/C⋯H = 8.2% and (*d*) H ⋯N/N⋯H = 5.5%. Since no considerable differences between the values were observed, the evaluations and graphics were performed for the structure with the *A*-labelled atoms only. The graphical representation of the Hirshfeld surface (*d*_norm_) is drawn in a figure with two separate opposite side-views of the mol­ecule with transparency and using a ball-and-stick model. The locations of the strongest inter­molecular contacts, *i.e*, the regions around the H1, H3 and S1 atoms (Fig. 4 ▸) are indicated in red. These atoms are those involved in the H⋯S inter­actions shown in the previous figures (Figs. 2 ▸ and 3 ▸). The contributions to the crystal packing are shown as two-dimensional Hirshfeld surface fingerprint plots (HSFP) with cyan dots (Fig. 5 ▸). The *d*_i_ (*x-*axis) and the *d*_e_ (*y-*axis) values are the closest inter­nal and external distances from given points on the Hirshfeld surface (in Å).

**Figure 6:**
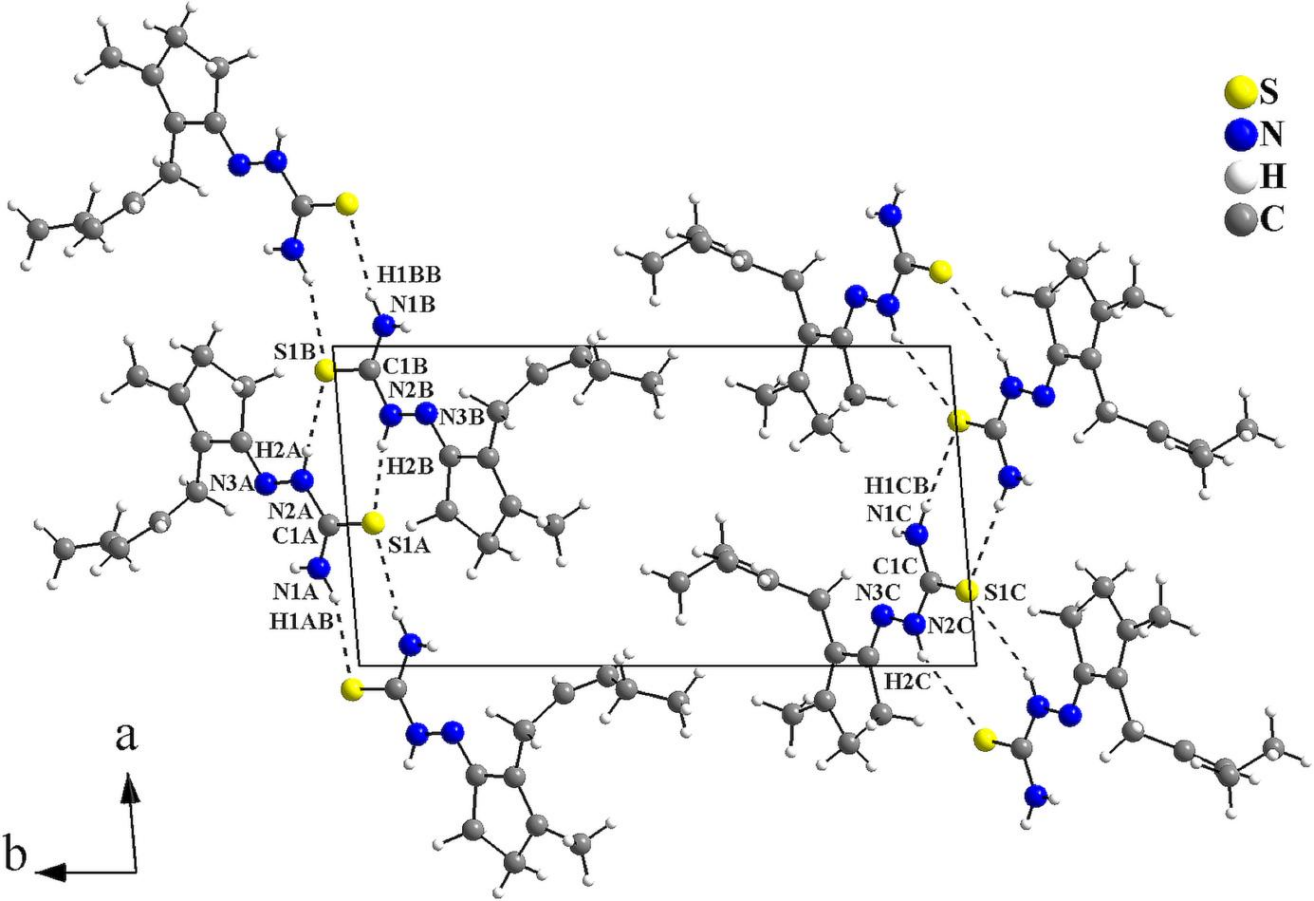
Crystal structure section of the α-*cis*-jasmone thio­semicarbazone (Orsoni *et al.*, 2020 ▸) viewed along [001]. Selected atoms of the **TSC** entities are labelled to indicate the three crystallographically independent mol­ecules [α-JATSC(A); α-JATSC(B); α-JATSC(C)]. The N—H⋯S inter­molecular inter­actions, forming rings with graph-set motif 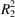 (8), are drawn as dashed lines and connect the mol­ecules into mono-periodic H-bonded ribbons along [100].

**Figure 7:**
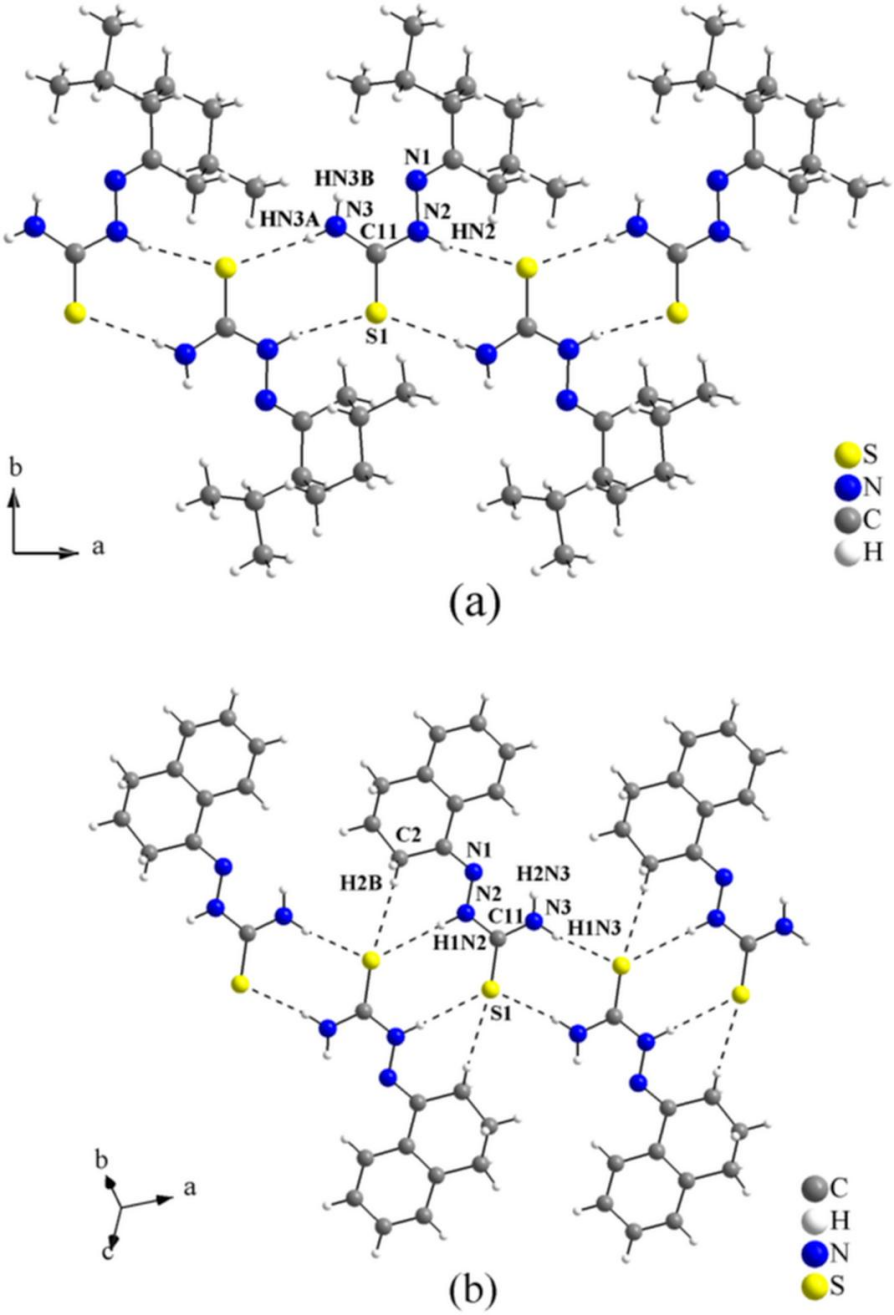
(*a*) (−)-Menthone thio­semicarbazone (Oliveira *et al.*, 2014 ▸) and (*b*) tetra­lone thio­semicarbazone (Oliveira *et al.*, 2012 ▸) graphical representations of the mono-periodic hydrogen-bonded ribbons structures along [100] and [1 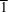 0], respectively. The mol­ecules are connected by H⋯S inter­molecular inter­actions drawn as dashed lines. The atoms of the **TSC** entities and one C—H donor in general positions are labelled. This packing pattern is common for non-substituted thio­semicarbazones attached to non-polar organic entities.

The crystal structure of the α-crystalline modification of the *cis*-jasmone thio­semicarbazone was reported recently (Orsoni *et al.*, 2020 ▸). As already mentioned above, the α-modification has three crystallographically independent mol­ecules in the asymmetric unit, namely α-JATSC(A), α-JATSC(B) and α-JATSC(C). In the crystal, the mol­ecules are connected by pairs of N—H⋯S inter­actions, with graph-set motif 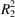 (8), into mono-periodic hydrogen-bonded ribbons along [100] (Fig. 6 ▸). The α-modification contains two crystallographically different strands. Within one of the strands, inversion centres are located at the centroids of every eight-membered C_2_H_2_N_2_S_2_ ring, while the other strand has no inter­nal symmetry. The β-modification has only one independent strand that has no inter­nal symmetry. For a comparison of selected geometric parameters of the α- and β-modifications of *cis*-jasmone thio­semicarbazone, see Table 2 ▸. The crystal structures of non-substituted thio­semicarbazones attached to non-polar organic groups have been studied by our group, such as the structures of the (−)-menthone (Oliveira *et al.*, 2014 ▸) and the tetra­lone thio­semicarbazone derivatives (Oliveira *et al.*, 2012 ▸, 2017 ▸). In the structure of the (−)-menthone thio­semicarbazone, the mol­ecules are linked by N—H⋯S inter­molecular inter­actions, forming rings with graph-set motif 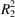 (8), into mono-periodic hydrogen-bonded ribbons along [100]. For the structure of the tetra­lone thio­ssemicarbazone, the mol­ecules are connected by N—H⋯S and C—H⋯S inter­molecular inter­actions along [1 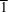 0], where rings of graph-set motifs 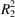 (8) and 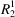 (7) are observed. The same supra­molecular arrangement was observed for both structures, forming a structural pattern for these entities (Fig. 7 ▸). This packing pattern is common for non-substituted thio­semicarbazones attached to non-polar organic entities, as observed in this work (Fig. 3 ▸).

## Synthesis and crystallization

The starting materials are commercially available and were used without further purification. The synthesis of *cis*-jasmone thio­semicarbazone was adapted from previously reported procedures (Freund & Schander, 1902 ▸; Oliveira *et al.*, 2017 ▸; Orsoni *et al.*, 2020 ▸). The mixture of ethano­lic solutions of *cis*-jasmone (8 mmol in 50 ml) and thio­semicarbazide (8 mmol in 50 ml), was catalysed with HCl and refluxed for 8 h. After cooling, the precipitated product was filtered off and washed with cold ethanol. Colourless single crystals suitable for X-ray diffraction were obtained from tetra­hydro­furan solution by slow evaporation of the solvent at room temperature. The template effect of the crystallization solvent and the temperature can be suggested as factors for the formation of the new crystalline modification of the *cis*-jasmone thio­semicarbazone, since the α-crystalline modification was crystallized from ethanol solution at 273 K (Orsoni *et al.*, 2020 ▸).

## Refinement

Crystal data, data collection and structure refinement details are summarized in Table 3 ▸. The mol­ecule of title compound shows disorder over the chain of the *cis*-jasmone fragment, namely the H8, C9 and C10 atoms (Fig. 1 ▸), which are *A*-labelled for the atoms with the higher s.o.f. value and *B*-labelled for the lower [site-occupancy ratio = 0.590 (14):0.410 (14)]. H atoms attached to the C2, C3, C6, C7, C11, N2 and N3 atoms were located in the difference Fourier map. The one bonded to N2 was refined freely, and those bonded to C2, C3, C6, C7, C11, and N3 were refined freely using the same isotropic displacement parameter for the atoms bonded to the same parent atom. The remaining hydrogen atoms were located in a difference-Fourier map, but were positioned with idealized geometry and refined isotropically using a riding model (HFIX command). Methyl H atoms were allowed to rotate but not to tip to best fit the experimental electron density. Thus, for the C10*A*H_3_ and C10*B*H_3_ fragments, with *U*_iso_(H) = 1.5 *U*_eq_(C), the C—H bond lengths were set to 0.96 Å. For the H atoms attached to the C8 atom and to the C9*A* and C9*B* atoms, with *U*_iso_(H) = 1.2 *U*_eq_(C), the C—H bond lengths were set to 0.93 and 0.97 Å, respectively.

## Supplementary Material

Crystal structure: contains datablock(s) I, publication_text. DOI: 10.1107/S2414314623010180/bt4143sup1.cif

Structure factors: contains datablock(s) I. DOI: 10.1107/S2414314623010180/bt4143Isup2.hkl

Supporting information file. DOI: 10.1107/S2414314623010180/bt4143Isup3.cml

CCDC reference: 2310189

Additional supporting information:  crystallographic information; 3D view; checkCIF report

## Figures and Tables

**Table 1:** Hydrogen-bond geometry (Å, °)

*D*—H⋯*A*	*D*—H	H⋯*A*	*D*⋯*A*	*D*—H⋯*A*
N2—H1⋯S1^i^	0.90 (3)	2.53 (3)	3.4142 (19)	166 (2)
N3—H3⋯S1^ii^	0.85 (3)	2.48 (3)	3.325 (2)	173 (3)
C2—H2*B*⋯S1^i^	1.00 (2)	2.93 (2)	3.436 (2)	112.2 (16)

Symmetry codes: (i) 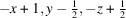 ; (ii) 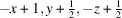 .

**Table 2:** Selected geometric parameters (Å, °) of the TSC entities for the *α*- and *β*-crystalline modifications of the *cis*-jasmone thio­semicarbazone *α*-JATSC(A), *α*-JATSC(B) and *α*-JATSC(C) refer to the three crystallographically independent mol­ecules in the *α*-crystalline modification of *cis*-jasmone thio­semicarbazone (Orsoni *et al.*, 2020 ▸) (Fig. 6 ▸). *β*-JATSC refers to the *β*-crystalline modification of *cis*-jasmone thio­semicarbazone reported in this work (Fig. 1 ▸).

	Bond length	N=N	N—C	C=S
Compound				
*α*-JATSC(A)		1.383 (5)	1.305 (5)	1.695 (5)
*α*-JATSC(B)		1.384 (5)	1.349 (5)	1.701 (5)
*α*-JATSC(C)		1.400 (5)	1.341 (5)	1.689 (5)
*β*-JATSC		1.388 (2)	1.345 (3)	1.698 (2)
				
	Atom chain 1	Torsion angle	Atom chain 2	Torsion angle
*α*-JATSC(A)	N3*A*—N2*A*—C1*A*—S1*A*	−179.4 (3)	N3*A*—N2*A*—C1*A*—N1*A*	0.0 (6)
*α*-JATSC(B)	N3*B*—N2*B*—C1*B*—S1*B*	180.0 (3)	N3*B*—N2*B*—C1*B*—N1*B*	0.2 (6)
*α*-JATSC(C)	N3*C*—N2*C*—C1*C*–S1*C*	177.4 (3)	N3*C*—N2*C*—C1*C*—N1*C*	−1.8 (6)
*β*-JATSC	N1—N2—C12—S1	174.04 (15)	N1—N2—C12—N3	−4.8 (3)

**Table 3:** Experimental details

Crystal data	
Chemical formula	C_12_H_19_N_3_S
*M*_r_	237.36
Crystal system, space group	Monoclinic, *P*2_1_/*c*
Temperature (K)	123
*a*, *b*, *c* (Å)	15.0159 (7), 8.0595 (3), 10.8243 (5)
β (°)	94.372 (3)
*V* (Å^3^)	1306.15 (10)
*Z*	4
Radiation type	Mo *K*α
μ (mm^−1^)	0.23
Crystal size (mm)	0.17 × 0.14 × 0.05

Data collection	
Diffractometer	Enraf–Nonius FR590 Kappa CCD
Absorption correction	Multi-scan (Blessing, 1995 ▸)
*T*_min_, *T*_max_	0.922, 0.998
No. of measured, independent and observed [*I* > 2σ(*I*)] reflections	24176, 3002, 2241
*R*_int_	0.083
(sin θ/λ)_max_ (Å^−1^)	0.651

Refinement	
*R*[*F* ^2^ > 2σ(*F* ^2^)], *wR*(*F* ^2^), *S*	0.054, 0.143, 1.09
No. of reflections	3002
No. of parameters	212
H-atom treatment	H atoms treated by a mixture of independent and constrained refinement
Δρ_max_, Δρ_min_ (e Å^−3^)	0.59, −0.45

Computer programs: *COLLECT* (Nonius, 1998 ▸), *HKL* *DENZO* and *SCALEPACK* (Otwinowski & Minor, 1997 ▸), *SIR92* (Altomare *et al.*, 1994 ▸), *SHELXL2018/3* (Sheldrick, 2015 ▸), *DIAMOND* (Brandenburg, 2006 ▸), CrystalExplorer (Wolff *et al.*, 2012 ▸), *WinGX* (Farrugia, 2012 ▸), *publCIF* (Westrip, 2010 ▸) and *enCIFer* (Allen *et al.*, 2004 ▸).

## full crystallographic data

### Crystal data

**Table:**

C_12_H_19_N_3_S	*F*(000) = 512
*M**_r_* = 237.36	*D*_x_ = 1.207 Mg m^−^^3^
Monoclinic, *P*2_1_/*c*	Mo *K*α radiation, λ = 0.71073 Å
*a* = 15.0159 (7) Å	Cell parameters from 60208 reflections
*b* = 8.0595 (3) Å	θ = 2.9–27.5°
*c* = 10.8243 (5) Å	µ = 0.23 mm^−^^1^
β = 94.372 (3)°	*T* = 123 K
*V* = 1306.15 (10) Å^3^	Plate, colourless
*Z* = 4	0.17 × 0.14 × 0.05 mm

### Data collection

**Table:**

Enraf–Nonius FR590 Kappa CCD diffractometer	3002 independent reflections
Radiation source: sealed X-ray tube, Enraf Nonius FR590	2241 reflections with *I* > 2σ(*I*)
Horizontally mounted graphite crystal monochromator	*R*_int_ = 0.083
Detector resolution: 9 pixels mm^-1^	θ_max_ = 27.6°, θ_min_ = 3.2°
CCD rotation images, thick slices, κ–goniostat scans	*h* = −19→19
Absorption correction: multi-scan (Blessing, 1995)	*k* = −10→10
*T*_min_ = 0.922, *T*_max_ = 0.998	*l* = −13→14
24176 measured reflections	

### Refinement

**Table:**

Refinement on *F*^2^	Primary atom site location: structure-invariant direct methods
Least-squares matrix: full	Secondary atom site location: difference Fourier map
*R*[*F*^2^ > 2σ(*F*^2^)] = 0.054	Hydrogen site location: mixed
*wR*(*F*^2^) = 0.143	H atoms treated by a mixture of independent and constrained refinement
*S* = 1.09	*w* = 1/[σ^2^(*F*_o_^2^) + (0.069*P*)^2^ + 0.7307*P*] where *P* = (*F*_o_^2^ + 2*F*_c_^2^)/3
3002 reflections	(Δ/σ)_max_ < 0.001
212 parameters	Δρ_max_ = 0.59 e Å^−^^3^
0 restraints	Δρ_min_ = −0.45 e Å^−^^3^

### Special details

**Table:**

Geometry. All esds (except the esd in the dihedral angle between two l.s. planes) are estimated using the full covariance matrix. The cell esds are taken into account individually in the estimation of esds in distances, angles and torsion angles; correlations between esds in cell parameters are only used when they are defined by crystal symmetry. An approximate (isotropic) treatment of cell esds is used for estimating esds involving l.s. planes.

### Fractional atomic coordinates and isotropic or equivalent isotropic displacement parameters (Å^2^)

**Table:**

	*x*	*y*	*z*	*U*_iso_*/*U*_eq_	Occ. (<1)
S1	0.49279 (4)	−0.38063 (6)	0.29692 (5)	0.02576 (19)	
N1	0.35988 (12)	−0.5052 (2)	−0.01509 (16)	0.0239 (4)	
N2	0.40985 (12)	−0.5194 (2)	0.09773 (17)	0.0235 (4)	
H1	0.4271 (17)	−0.617 (3)	0.133 (2)	0.030 (7)*	
N3	0.42480 (14)	−0.2376 (2)	0.09074 (19)	0.0275 (5)	
H2	0.3988 (18)	−0.242 (4)	0.016 (3)	0.039 (5)*	
H3	0.4455 (19)	−0.149 (4)	0.126 (3)	0.039 (5)*	
C1	0.32864 (14)	−0.6392 (3)	−0.0662 (2)	0.0224 (5)	
C2	0.33976 (16)	−0.8181 (3)	−0.0266 (2)	0.0251 (5)	
H2A	0.3276 (15)	−0.833 (3)	0.065 (2)	0.024 (4)*	
H2B	0.4031 (17)	−0.853 (3)	−0.036 (2)	0.024 (4)*	
C3	0.27458 (17)	−0.9129 (3)	−0.1170 (2)	0.0295 (5)	
H3A	0.3041 (16)	−1.002 (4)	−0.156 (2)	0.035 (5)*	
H3B	0.2234 (17)	−0.961 (3)	−0.074 (2)	0.035 (5)*	
C4	0.24066 (15)	−0.7858 (3)	−0.2109 (2)	0.0269 (5)	
C5	0.27121 (15)	−0.6322 (3)	−0.1810 (2)	0.0253 (5)	
C6	0.25021 (17)	−0.4704 (3)	−0.2465 (2)	0.0299 (5)	
H6A	0.3013 (19)	−0.413 (4)	−0.246 (3)	0.039 (5)*	
H6B	0.2284 (17)	−0.490 (3)	−0.337 (3)	0.039 (5)*	
C7	0.18173 (18)	−0.3701 (3)	−0.1851 (2)	0.0349 (6)	
H7	0.1977 (18)	−0.347 (3)	−0.097 (3)	0.041 (8)*	
C8	0.1055 (2)	−0.3144 (4)	−0.2354 (3)	0.0520 (8)	
H8A	0.077777	−0.233393	−0.190673	0.062*	0.590 (14)
H8B	0.059808	−0.292155	−0.184493	0.062*	0.410 (14)
C9A	0.0569 (4)	−0.3651 (9)	−0.3570 (6)	0.0381 (17)	0.590 (14)
H9A1	0.014033	−0.451696	−0.342863	0.046*	0.590 (14)
H9A2	0.099214	−0.408217	−0.412357	0.046*	0.590 (14)
C10A	0.0088 (6)	−0.2146 (8)	−0.4159 (9)	0.0420 (18)	0.590 (14)
H10A	−0.034413	−0.174691	−0.362002	0.063*	0.590 (14)
H10B	−0.020921	−0.245795	−0.494084	0.063*	0.590 (14)
H10C	0.051400	−0.128624	−0.428493	0.063*	0.590 (14)
C11	0.17972 (19)	−0.8356 (4)	−0.3194 (3)	0.0371 (6)	
H11A	0.161 (2)	−0.743 (4)	−0.376 (3)	0.051 (5)*	
H11B	0.125 (2)	−0.877 (4)	−0.291 (3)	0.051 (5)*	
H11C	0.206 (2)	−0.916 (4)	−0.370 (3)	0.051 (5)*	
C12	0.43977 (14)	−0.3783 (2)	0.1527 (2)	0.0221 (4)	
C9B	0.0882 (7)	−0.2831 (15)	−0.3810 (7)	0.043 (3)	0.410 (14)
H9B1	0.108084	−0.172234	−0.400126	0.052*	0.410 (14)
H9B2	0.120614	−0.362544	−0.427766	0.052*	0.410 (14)
C10B	−0.0104 (10)	−0.290 (3)	−0.4108 (15)	0.090 (6)	0.410 (14)
H10D	−0.034871	−0.381309	−0.367063	0.135*	0.410 (14)
H10E	−0.022841	−0.306245	−0.498360	0.135*	0.410 (14)
H10F	−0.037061	−0.188515	−0.386266	0.135*	0.410 (14)

### Atomic displacement parameters (Å^2^)

**Table:**

	*U*^11^	*U*^22^	*U*^33^	*U*^12^	*U*^13^	*U*^23^
S1	0.0399 (4)	0.0149 (3)	0.0215 (3)	−0.0017 (2)	−0.0048 (2)	−0.0004 (2)
N1	0.0299 (10)	0.0203 (9)	0.0208 (9)	−0.0005 (8)	−0.0020 (7)	0.0003 (7)
N2	0.0330 (10)	0.0148 (9)	0.0215 (9)	−0.0010 (7)	−0.0045 (8)	−0.0010 (7)
N3	0.0414 (12)	0.0160 (9)	0.0241 (11)	−0.0025 (8)	−0.0047 (9)	−0.0005 (8)
C1	0.0252 (11)	0.0207 (11)	0.0215 (11)	−0.0015 (8)	0.0023 (8)	−0.0016 (9)
C2	0.0298 (12)	0.0174 (10)	0.0275 (12)	−0.0013 (9)	−0.0023 (9)	−0.0026 (9)
C3	0.0343 (13)	0.0249 (12)	0.0289 (12)	−0.0053 (10)	−0.0004 (10)	−0.0040 (10)
C4	0.0252 (11)	0.0303 (12)	0.0250 (11)	−0.0026 (9)	0.0014 (9)	−0.0039 (10)
C5	0.0265 (11)	0.0266 (11)	0.0226 (11)	0.0005 (9)	0.0009 (9)	−0.0002 (9)
C6	0.0339 (13)	0.0299 (12)	0.0253 (12)	0.0003 (10)	−0.0026 (10)	0.0028 (10)
C7	0.0506 (16)	0.0272 (13)	0.0261 (13)	0.0051 (11)	−0.0020 (11)	−0.0040 (10)
C8	0.0579 (19)	0.0569 (18)	0.0405 (16)	0.0247 (15)	−0.0007 (14)	−0.0123 (15)
C9A	0.032 (3)	0.033 (3)	0.048 (3)	0.003 (2)	−0.003 (2)	−0.004 (3)
C10A	0.041 (4)	0.038 (3)	0.046 (4)	0.006 (2)	−0.007 (3)	0.003 (3)
C11	0.0353 (15)	0.0436 (15)	0.0316 (14)	−0.0105 (12)	−0.0024 (11)	−0.0053 (12)
C12	0.0258 (11)	0.0156 (10)	0.0249 (11)	0.0009 (8)	0.0019 (9)	−0.0006 (9)
C9B	0.047 (5)	0.046 (5)	0.037 (4)	0.004 (4)	0.003 (3)	0.005 (3)
C10B	0.047 (7)	0.173 (19)	0.050 (6)	0.012 (10)	0.001 (5)	0.021 (11)

### Geometric parameters (Å, º)

**Table:**

S1—C12	1.698 (2)	C7—C8	1.308 (4)
N1—C1	1.285 (3)	C7—H7	0.98 (3)
N1—N2	1.388 (2)	C8—C9A	1.512 (6)
N2—C12	1.345 (3)	C8—C9B	1.598 (8)
N2—H1	0.90 (3)	C8—H8A	0.9300
N3—C12	1.328 (3)	C8—H8B	0.9300
N3—H2	0.87 (3)	C9A—C10A	1.526 (10)
N3—H3	0.85 (3)	C9A—H9A1	0.9700
C1—C5	1.458 (3)	C9A—H9A2	0.9700
C1—C2	1.510 (3)	C10A—H10A	0.9600
C2—C3	1.534 (3)	C10A—H10B	0.9600
C2—H2A	1.03 (2)	C10A—H10C	0.9600
C2—H2B	1.00 (2)	C11—H11A	1.00 (3)
C3—C4	1.504 (3)	C11—H11B	0.96 (3)
C3—H3A	0.96 (3)	C11—H11C	0.95 (3)
C3—H3B	1.00 (3)	C9B—C10B	1.49 (2)
C4—C5	1.351 (3)	C9B—H9B1	0.9700
C4—C11	1.489 (3)	C9B—H9B2	0.9700
C5—C6	1.506 (3)	C10B—H10D	0.9600
C6—C7	1.502 (4)	C10B—H10E	0.9600
C6—H6A	0.90 (3)	C10B—H10F	0.9600
C6—H6B	1.02 (3)		
			
C1—N1—N2	117.67 (18)	C7—C8—C9B	122.4 (4)
C12—N2—N1	117.32 (18)	C7—C8—H8A	115.9
C12—N2—H1	118.2 (16)	C9A—C8—H8A	115.9
N1—N2—H1	124.3 (16)	C7—C8—H8B	118.8
C12—N3—H2	118.8 (19)	C9B—C8—H8B	118.8
C12—N3—H3	116.3 (19)	C8—C9A—C10A	109.3 (5)
H2—N3—H3	125 (3)	C8—C9A—H9A1	109.8
N1—C1—C5	120.47 (19)	C10A—C9A—H9A1	109.8
N1—C1—C2	130.6 (2)	C8—C9A—H9A2	109.8
C5—C1—C2	108.90 (18)	C10A—C9A—H9A2	109.8
C1—C2—C3	104.07 (18)	H9A1—C9A—H9A2	108.3
C1—C2—H2A	111.4 (14)	C9A—C10A—H10A	109.5
C3—C2—H2A	113.8 (13)	C9A—C10A—H10B	109.5
C1—C2—H2B	108.9 (13)	H10A—C10A—H10B	109.5
C3—C2—H2B	111.0 (13)	C9A—C10A—H10C	109.5
H2A—C2—H2B	107.6 (19)	H10A—C10A—H10C	109.5
C4—C3—C2	105.00 (19)	H10B—C10A—H10C	109.5
C4—C3—H3A	110.8 (15)	C4—C11—H11A	114.3 (18)
C2—C3—H3A	111.1 (15)	C4—C11—H11B	109.3 (18)
C4—C3—H3B	110.1 (15)	H11A—C11—H11B	104 (2)
C2—C3—H3B	111.7 (14)	C4—C11—H11C	112.3 (18)
H3A—C3—H3B	108 (2)	H11A—C11—H11C	105 (3)
C5—C4—C11	127.8 (2)	H11B—C11—H11C	111 (3)
C5—C4—C3	111.8 (2)	N3—C12—N2	117.47 (19)
C11—C4—C3	120.4 (2)	N3—C12—S1	121.54 (17)
C4—C5—C1	109.67 (19)	N2—C12—S1	120.98 (16)
C4—C5—C6	128.7 (2)	C10B—C9B—C8	107.0 (10)
C1—C5—C6	121.56 (19)	C10B—C9B—H9B1	107.9
C7—C6—C5	112.6 (2)	C8—C9B—H9B1	109.0
C7—C6—H6A	109.3 (18)	C10B—C9B—H9B2	113.0
C5—C6—H6A	107.2 (18)	C8—C9B—H9B2	111.1
C7—C6—H6B	109.0 (15)	H9B1—C9B—H9B2	108.7
C5—C6—H6B	111.1 (16)	C9B—C10B—H10D	109.5
H6A—C6—H6B	108 (2)	C9B—C10B—H10E	109.5
C8—C7—C6	127.4 (2)	H10D—C10B—H10E	109.5
C8—C7—H7	118.7 (16)	C9B—C10B—H10F	109.5
C6—C7—H7	113.9 (16)	H10D—C10B—H10F	109.5
C7—C8—C9A	128.2 (3)	H10E—C10B—H10F	109.5
			
C1—N1—N2—C12	−177.82 (19)	C2—C1—C5—C4	4.5 (3)
N2—N1—C1—C5	176.34 (19)	N1—C1—C5—C6	3.0 (3)
N2—N1—C1—C2	−2.8 (4)	C2—C1—C5—C6	−177.7 (2)
N1—C1—C2—C3	171.8 (2)	C4—C5—C6—C7	100.2 (3)
C5—C1—C2—C3	−7.4 (2)	C1—C5—C6—C7	−77.0 (3)
C1—C2—C3—C4	7.3 (3)	C5—C6—C7—C8	−123.5 (3)
C2—C3—C4—C5	−5.1 (3)	C6—C7—C8—C9A	14.5 (7)
C2—C3—C4—C11	175.9 (2)	C6—C7—C8—C9B	−24.3 (7)
C11—C4—C5—C1	179.4 (2)	C7—C8—C9A—C10A	−145.8 (5)
C3—C4—C5—C1	0.5 (3)	N1—N2—C12—N3	−4.8 (3)
C11—C4—C5—C6	1.9 (4)	N1—N2—C12—S1	174.04 (15)
C3—C4—C5—C6	−177.1 (2)	C7—C8—C9B—C10B	156.7 (12)
N1—C1—C5—C4	−174.7 (2)		

### Hydrogen-bond geometry (Å, º)

**Table:**

*D*—H···*A*	*D*—H	H···*A*	*D*···*A*	*D*—H···*A*
N2—H1···S1^i^	0.90 (3)	2.53 (3)	3.4142 (19)	166 (2)
N3—H3···S1^ii^	0.85 (3)	2.48 (3)	3.325 (2)	173 (3)
C2—H2*B*···S1^i^	1.00 (2)	2.93 (2)	3.436 (2)	112.2 (16)

Symmetry codes: (i) −*x*+1, *y*−1/2, −*z*+1/2; (ii) −*x*+1, *y*+1/2, −*z*+1/2.
